# Setback zones can effectively reduce exposure to sea-level rise in Europe

**DOI:** 10.1038/s41598-023-32059-9

**Published:** 2023-04-04

**Authors:** Claudia Wolff, Hedda Bonatz, Athanasios T. Vafeidis

**Affiliations:** Coastal Risks and Sea‑Level Rise Research Group, Department of Geography, Christian-Albrechts University Kiel, Kiel, Germany

**Keywords:** Natural hazards, Environmental sciences, Environmental impact

## Abstract

Coastal space is one of the most valuable assets of the EU coastal member states, as the coast is highly urbanized. Hard engineering has traditionally been employed to protect communities in coastal lowlands, but as this alternative becomes less sustainable and more costly, coastal managers are increasingly turning to landuse planning strategies, such as setback zones or managed retreat. To explore the efficiency of these planning tools in reducing future urban exposure to sea-level rise and associated hazards, we developed spatially explicit projections of urban extent that account for different socio-economic futures and various types of setback zones. We find that the establishment of coastal setback zones can reduce the exposure of new urban development by at least 50% in the majority of EU countries by 2100. Our results emphasize that future urban exposure to sea-level rise will be significantly influenced by the ways in which we plan, design, and develop urban space in the EU coastal lowlands.

## Introduction

A large determinant of future coastal risk is related to the choice of where to build and where to live, and not only to changes in extreme events or sea-level rise due to a warming climate^1^. The research community agrees that the characteristics of coastal floods are changing as a result of ocean thermal expansion and the melting of land-ice masses, which is leading to rising sea levels worldwide^2^. Additionally, low atmospheric pressure and storms that may become stronger could further influence coastal flooding and make it more severe.

At the same time, the population and economic activities of the EU are expected to change as well. Currently, more than 200 million EU citizens are living within a distance of 50 km from the coast^3^, while migration towards the coast and from rural to urban areas seems to be continuing^4^, and urban development is evolving accordingly. Population densification, in the form of urban development or urbanization, affects coastal exposure and potentially vulnerability^5,6^. Further, the observed increase in land uptake due to increases in population and especially wealth per person in recent decades^7^ has stimulated the demand for housing^8^. According to Gao and O’Neill^8^, the number of people per area of built-up land has continually decreased during the past several decades, and this trend is anticipated to continue in the upcoming decades, resulting in an increase in built-up land all over the world. Changes in the spatial patterns, rate and type of urban development will therefore determine future coastal flood risk to population and infrastructure, as a flood is only associated with risk to humans if there is property (e.g. built environment or critical infrastructure) and people in its way.

Different adaptation options, namely protection, accommodation, retreat, or advancement^9^, exist to reduce coastal risk and help to protect urban settlements from coastal flooding. Most of these options are designed to protect people and infrastructure that are already in place in flood-prone areas. Traditionally, hard structures, such as dikes and seawalls, have been the main coastal adaptation strategy for urban areas, which allow for urban fabric and communities to grow along the coast. This form of protection offers advantages for the economy and creates societal benefits. However, the disadvantages are that it attracts increased investments and locks coastal communities into a position of continuously maintaining and upgrading coastal defences^10^, while simultaneously increasing residual risk^11^ as protection may fail.

Landuse planning can therefore be an effective way to protect and maintain coastlines by influencing where people will live in the future. Restricting new development in potentially exposed coastal regions could help to lower future flood risk upfront. Some Mediterranean EU countries have implemented such legislation, called coastal setback zones^12^, which is prescribed in the Integrated Coastal Zone Management (ICZM) protocol of the Barcelona Convention^13^. The ICZM Protocol prescribes the implementation of a 100 m setback zone, where further development is restricted^14^. Another example of the implementation of coastal landuse regulation in the EU can be found in Romania. According to Romanian law, permanent structures are not permitted in the "protected area" along the coast, which may be 50–150 m wide^12^. To our knowledge, setback zones have so far only been implemented based on a specific distance from the coast, there are no examples of setback zones based on other parameters, such as elevation.

Not much research has been done to quantify the potential of setback zones in reducing future coastal exposure in Europe^15^, even though several studies highlight that one of the most effective measures is to restrict urban development inside the coastal floodplain^16–18^. Our work seeks to deepen the understanding of the potential of different coastal setback zones in the EU by: (1) assessing the avoided urban exposure due to the implementation of setback zones under various socioeconomic scenarios (using the global Shared Socioeconomic Pathways, SSPs^19^); (2) provide country-specific information on which types of setback zones are the most beneficial in reducing urban exposure; and (3) estimate the total urban extent (corresponding to the artificial surface class code 1 from CORINE that includes residential, industrial, commercial, transport, and green urban areas; see methods section for more details) within different setback zones that are potentially exposed, to provide an estimate of the current coastal retreat prospect within Europe.

To address the above questions, we have developed spatially-explicit urban extent projections for all coastal EU Member States (plus Bosnia and Herzegovina (BIH), Great-Britain (GBR), and Norway (NOR)), employing the urban model developed by Wolff et al. 2020^18^. We extend the model by integrating different coastal setback zones into the projections. The implementation of different setback zones allows us to assess their potential effectiveness in reducing urban exposure and provides insights into possible solutions applicable for spatial planning in different local settings.

## Results

### The EU coastal lowlands are currently highly urbanized

**Figure 1:**
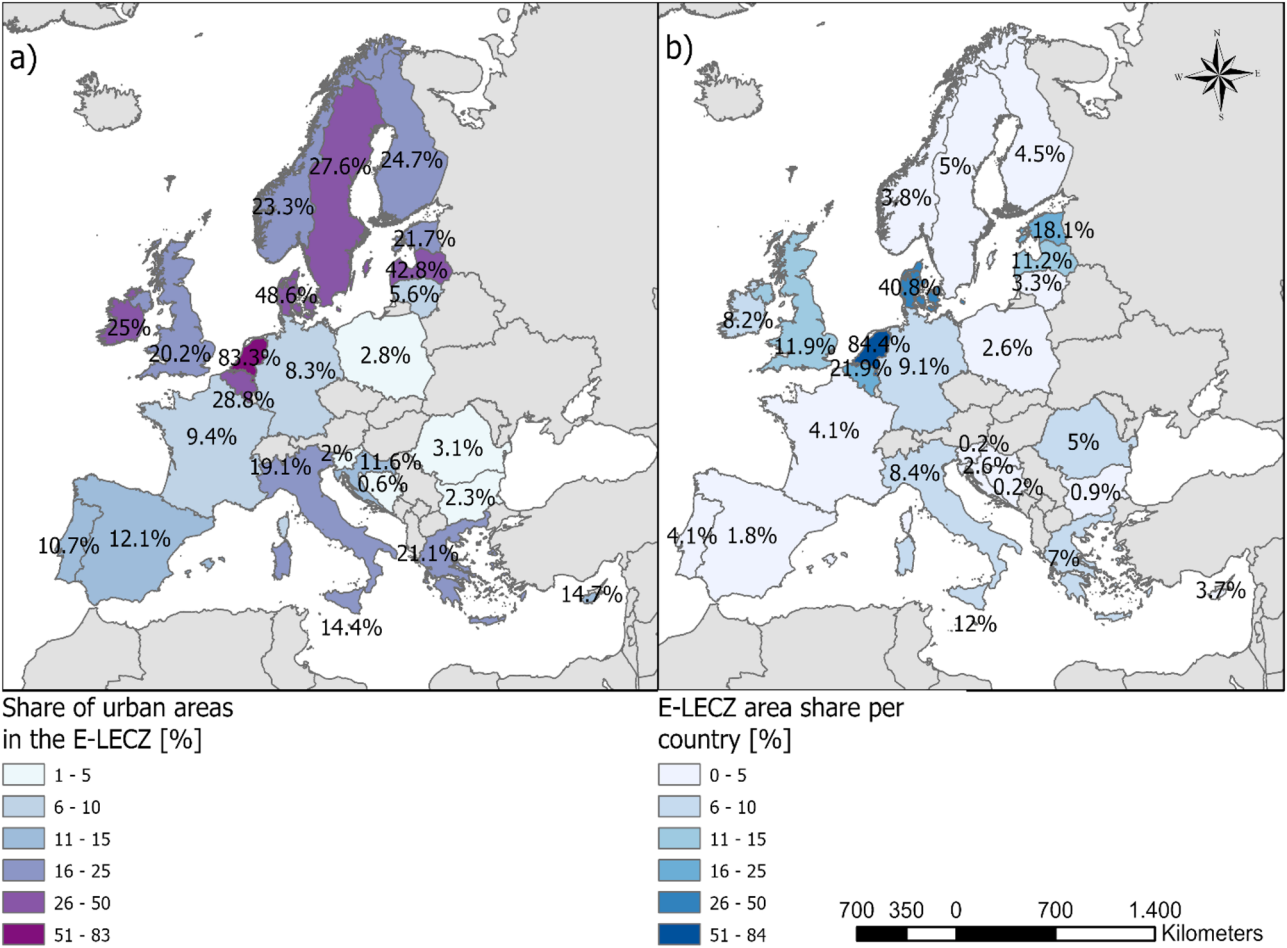
Coastal EU member states’ (plus BIH, GBR, and NOR) (**a**) share of urban areas in the E-LECZ [based on CORINE2012] and (**b**) share of the E-LECZ area [based on MERIT]. Darker colors indicate higher percentages. Grey countries are not included in the study.

We analyse the area below 20 m and hydrologically connected to the ocean (called Extended Low-Elevation Coastal Zone = E-LECZ in the remainder) to assess changes in coastal urban exposure. We opted for the E-LECZ as it encompasses the wider coastal lowlands and accounts for practically any plausible scenario of potential future changes in mean sea level, associated extreme water levels, including high-end scenarios with a low probability of occurrence but very high impact potential; as well as to account for data-related uncertainties in calculating coastal flood exposure (e.g., stemming from uncertainties inherent in surface elevation datasets such as MERIT^20,21^). We find that 6.3% of the land of all EU coastal countries is within the E-LECZ, assuming no adaptation measures are in place. This coastal strip (E-LECZ) currently contains 15.14% of the total urban area in the coastal EU Member States (plus BIH, GBR, and NOR), which indicates a high density of urban fabric in the EU coastal lowlands (see Fig. 1). In Norway, for example, the proportion of the E-LECZ is 3.8%, which comprises 23.3% of the country's total urban area. Similarly, in Spain and Greece, the E-LECZ accounts for 1.8% and 7% of the country’s total area, respectively, and hosts 12% and 21% of the country's urban areas. The three countries with the highest E-LECZ area share are the Netherlands (84%), Denmark (41%) and Belgium (22%). The Netherlands and Denmark are also the top countries in terms of urban area share in the E-LECZ, with 83% and 49%, respectively. Latvia ranks third with 43% of the urban area in the E-LECZ. In summary, in the majority of EU countries, the proportion of urban areas in the E-LECZ is significantly higher than the proportion of E-LECZ areas in the respective country (see Fig. 1).

### The future extent of urban areas (inland and coastal) in Europe can vary considerably by 2100, depending on future socioeconomic conditions

**Figure 2:**
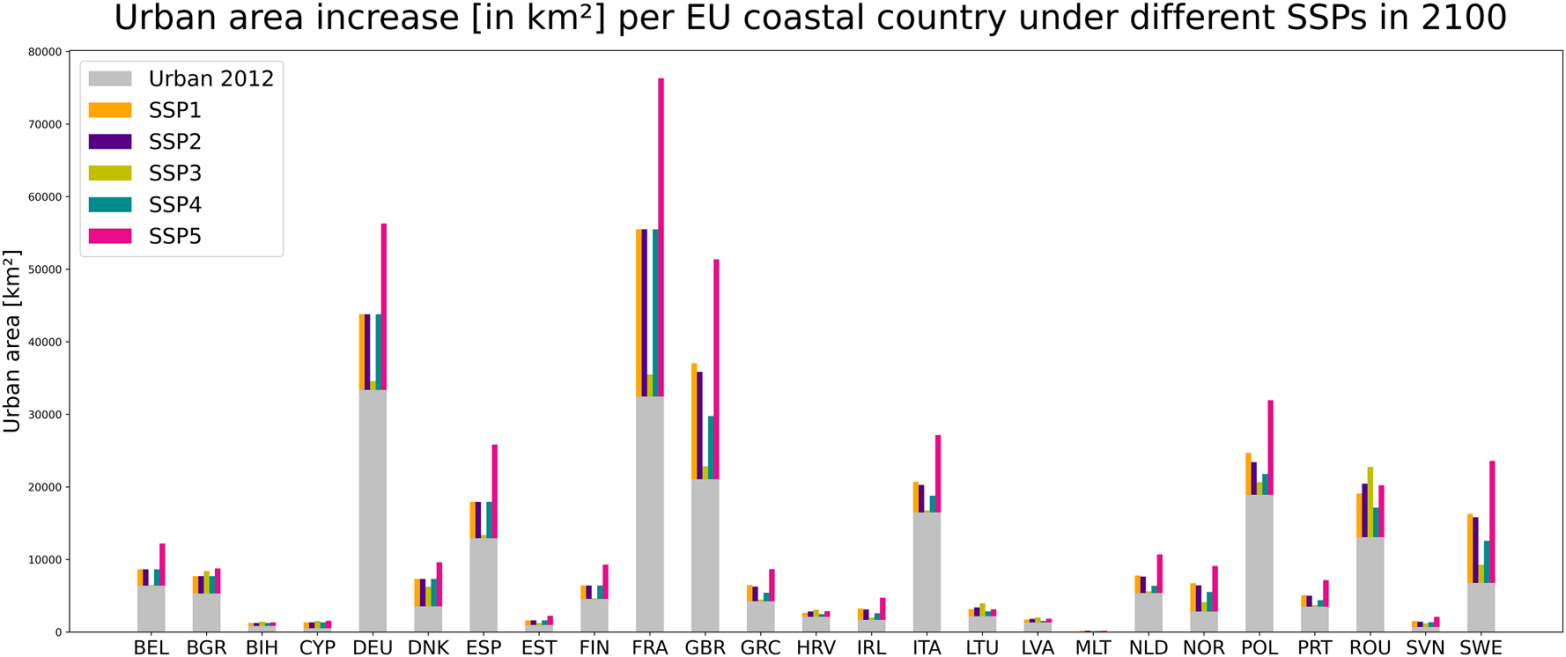
Urban extent [km^2^] per EU coastal country under different SSPs in 2100.

According to our projections, the total amount of urban land in all coastal EU Member States (plus BIH, GBR, and NOR) by 2100 will be between 236,055 and 408,243 km^2^ depending on the SSP. Comparing this value to 201,249 km^2^ in 2012, the urban area could grow between 17 and 102% by 2100. The total extent of urban areas is expected to increase the most in France (76,322 km^2^), followed by Germany (56,293 km^2^) and Great Britain (51,348 km^2^) under SSP5 (Fossil-fueled development) (see Fig. 2 and Table 1). SSP5 leads to the highest urban extent in most countries. Exceptions are Bosnia and Herzegovina, Latvia, Croatia, Romania, and Lithuania, where SSP3 (Regional rivalry) leads to the highest urban extent, and Malta, where SSP2 (Middle of the road) produces the highest numbers (see Table 1). The reason for SSP5 being the high growth scenario in most countries is mainly related to the rapid population and GDP growth under SSP5 in Europe^22^. Low fertility rates and population decline in the majority of the developed world are characteristics of SSP3^23^, which will result in slow urban expansion in the wealthier European OECD (Organisation for Economic Co-operation and Development) countries (e.g. France, Greece, Italy, Slovenia), leading to the lowest growth in those countries. On the other hand, in the same scenario, low-income countries such as Bosnia and Herzegovina, Latvia, Croatia, Romania, and Lithuania generate the highest urban extent as a result of their rapid population growth under SSP3.

**Table 1:**
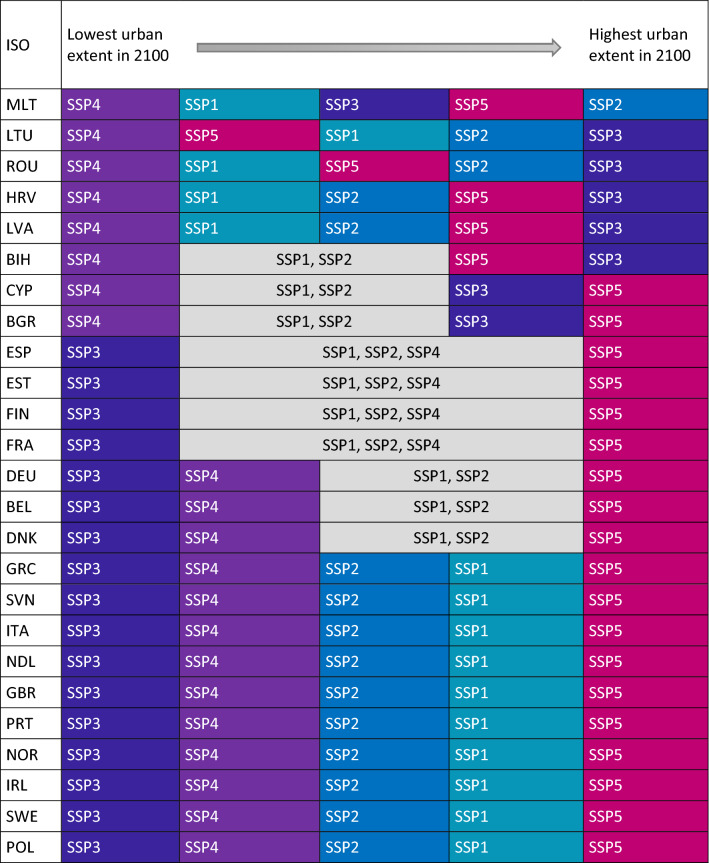
SSP ranging from the lowest (left) to the highest (right) urban extent for all countries in 2100 [without setback zones]. In case some SSPs produced nearly the same urban extent, we colored the columns grey.

**Figure 3:**
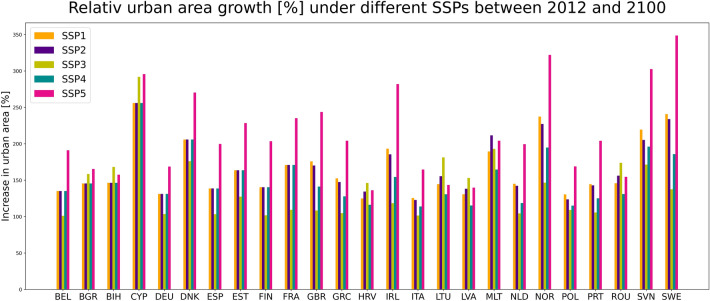
Increase in relative urban extent under the five different SSPs in all EU coastal countries between 2012 and 2100.

A different picture emerges when utilizing relative measures (i.e., change rates) as opposed to absolute measures (i.e., amounts of changes) (please see Fig. 3). In relative terms: the extent of urban areas will at least double in all EU coastal countries by 2100 (compared to 2012). Sweden stands out, with an increase in the extent of urban areas of up to 348% under SSP5, followed by Norway (322%) and Slovenia (308%). These results suggest a high demand for land for urban development in those countries.

Our results also suggest that scenarios with different driving forces can result in similar extents of urban areas (see Figs. 2 and 3, and Table 1). For instance, SSP1 (Sustainability), SSP2 (Middle of the road), and SSP4 (Inequality) produce similar urban areas in many countries in 2100 even though the underlying drivers are different. SSP1 is characterized by a high rate of urbanization; well-managed (compact) urban spatial patterns; low global population growth; medium fertility in richer OECD countries; and high-income growth but slower long-term economic growth. SSP2 follows historical spatial patterns and is characterized by central urbanization rates, moderate population and medium income growth. High rates of urbanization in high- and middle-income countries, mixed urban spatial patterns, and slow rates of population and income growth in EU member states are all characteristics of SSP4^24–26^. These findings demonstrate that the population-income-urban-growth relationship is very heterogeneous in the coastal EU Member States.

### Land use planning will substantially influence future coastal urban exposure to sea-level rise and associated hazards in Europe

#### Coastal urban exposure without the implementation of setback zones

**Figure 4:**
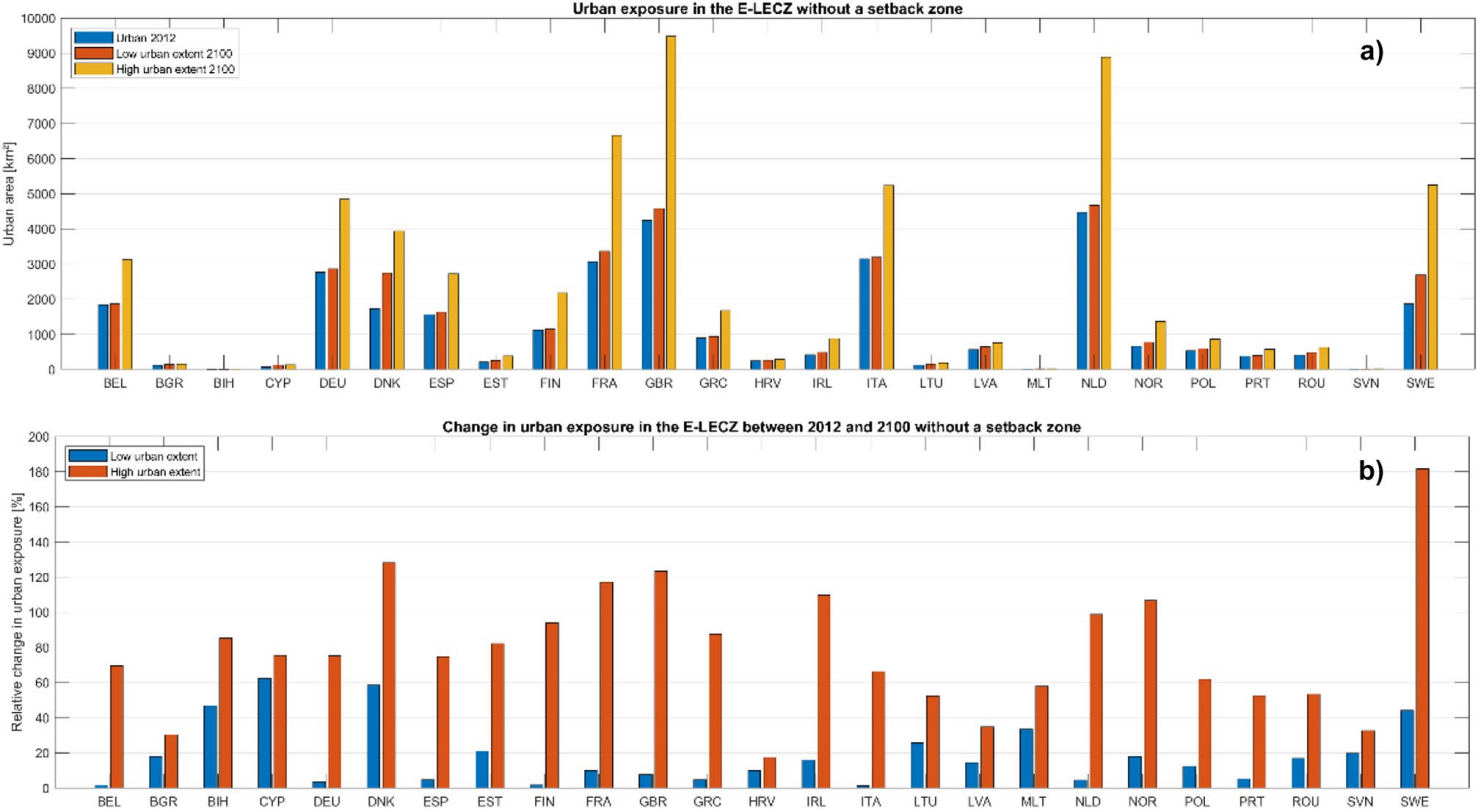
Urban exposure in the E-LECZ without the implementation of a coastal setback zone. (**a**) Shows the absolute values of urban areas in the E-LECZ in 2012 as well as the potential urban extent under a low and high urban growth scenario in 2100. (b) Illustrates the relative change in urban exposure in the E-LECZ under a low and high urban growth scenario between 2012 and 2100. Please note: Y-scale is changing between the panels.

Future urban exposure to sea-level rise in the E-LECZ could potentially rise by up to 182% under a high urban growth scenario within a country (see Fig. 4b—Sweden; more information about the high urban growth scenarios can be found in the methods section), without the implementation of a coastal setback zone. In absolute terms, Great Britain, the Netherlands, and France will have the highest coastal exposure of urban areas in 2100 (see Fig. 4a), illustrating that coastal urban exposure in Europe is highest in nations with a long coastline and large coastal floodplain (see Figs. 1, 4a, and b). The projected urban extent within the coastal floodplain varies significantly across the different scenarios (see Fig. 4b). The increase in urban exposure from 2012 to 2100 in the Netherlands, Italy, Belgium, Great Britain, Finland, Norway, France, Greece, Ireland, Germany, Spain, and Sweden shows the strong influence of the urban growth scenario, demonstrating that future urban land demand will generally drive future coastal urban exposure in the EU.

#### Coastal urban exposure under different setback zones

**Figure 5:**
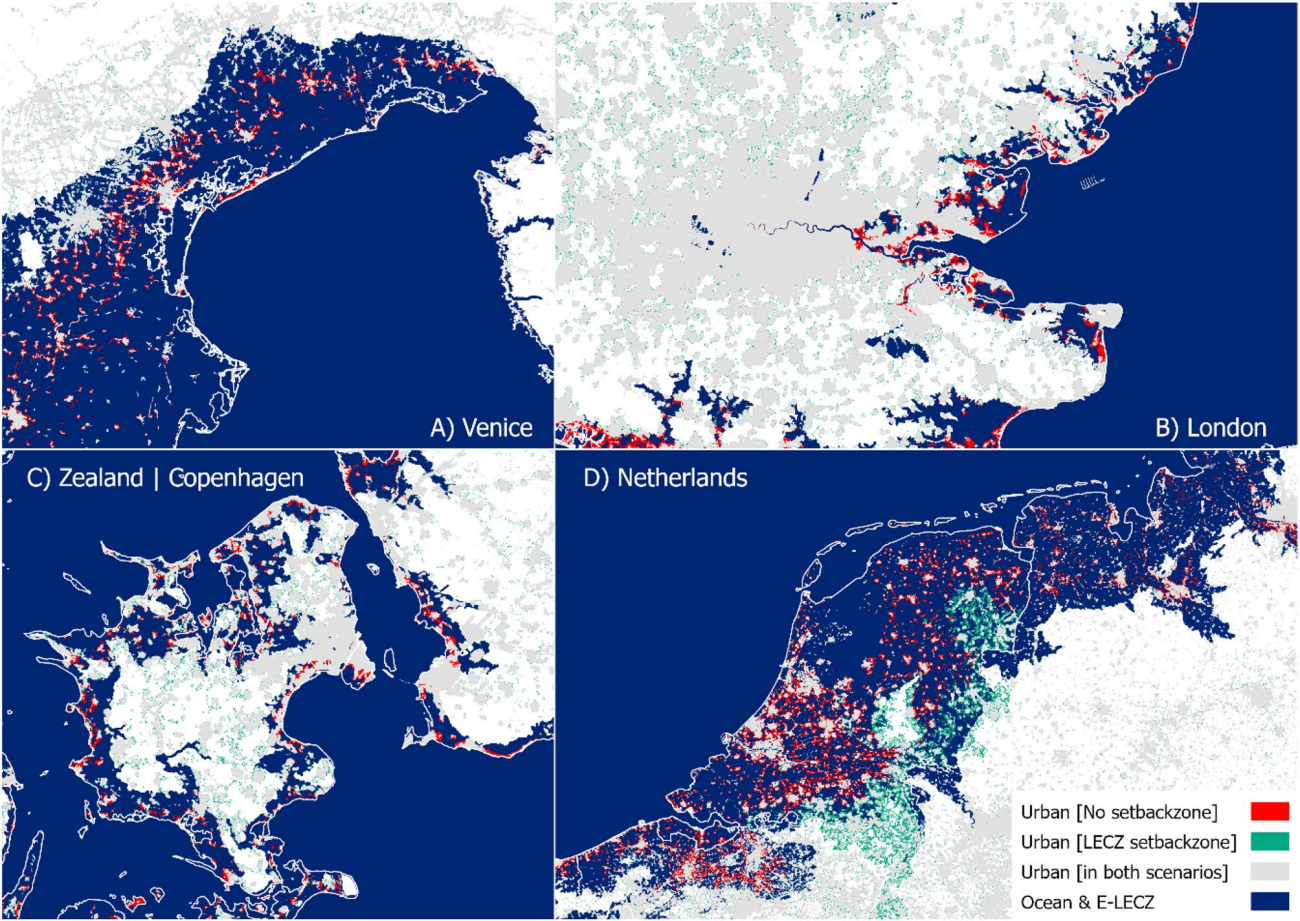
Comparison of the spatial allocation of new urban fabric under a 10 m elevation setback (called LECZ setback) and No setback in 2100 [high urban growth scenario].

On a European level, our urban extent projections are the first to include coastal setback zones in a spatially explicit manner with a 100 m resolution. Figure 5 shows how urban extent varies spatially if a setback zone of below 10 m of eleveation is implemented, compared to a no setback zone scenario, in four different locations within Europe in 2100.

**Figure 6:**
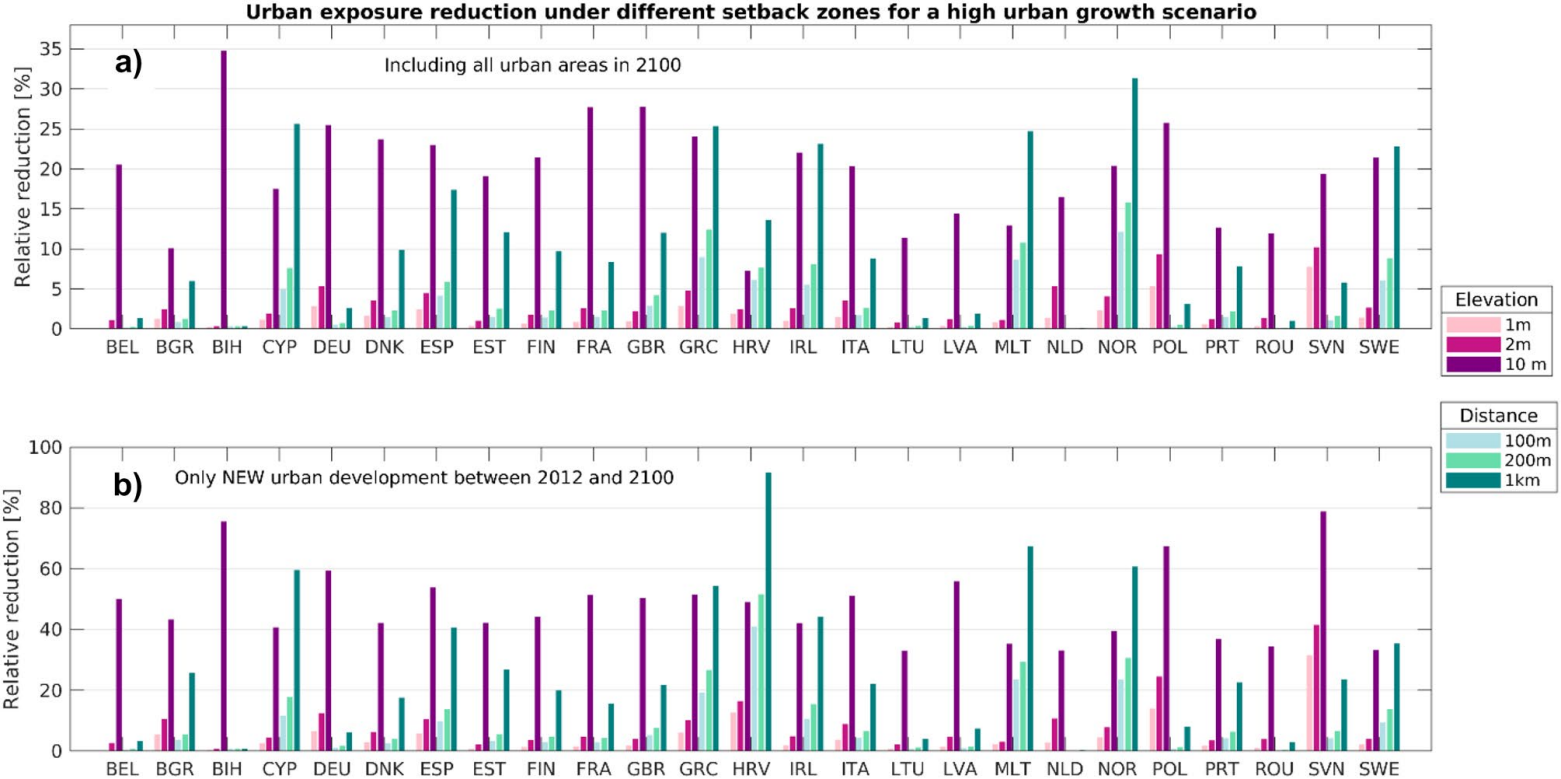
How effective are setback zones in reducing future urban exposure in the E-LECZ. (**a**) Relative reduction in overall urban exposure in the E-LECZ under different setback zones in 2100. (**b**) Relative reduction in urban exposure of new urban development between 2012 and 2100 [high urban growth scenario]. Please note: Y-scale is changing between the panels.

**Figure 7:**
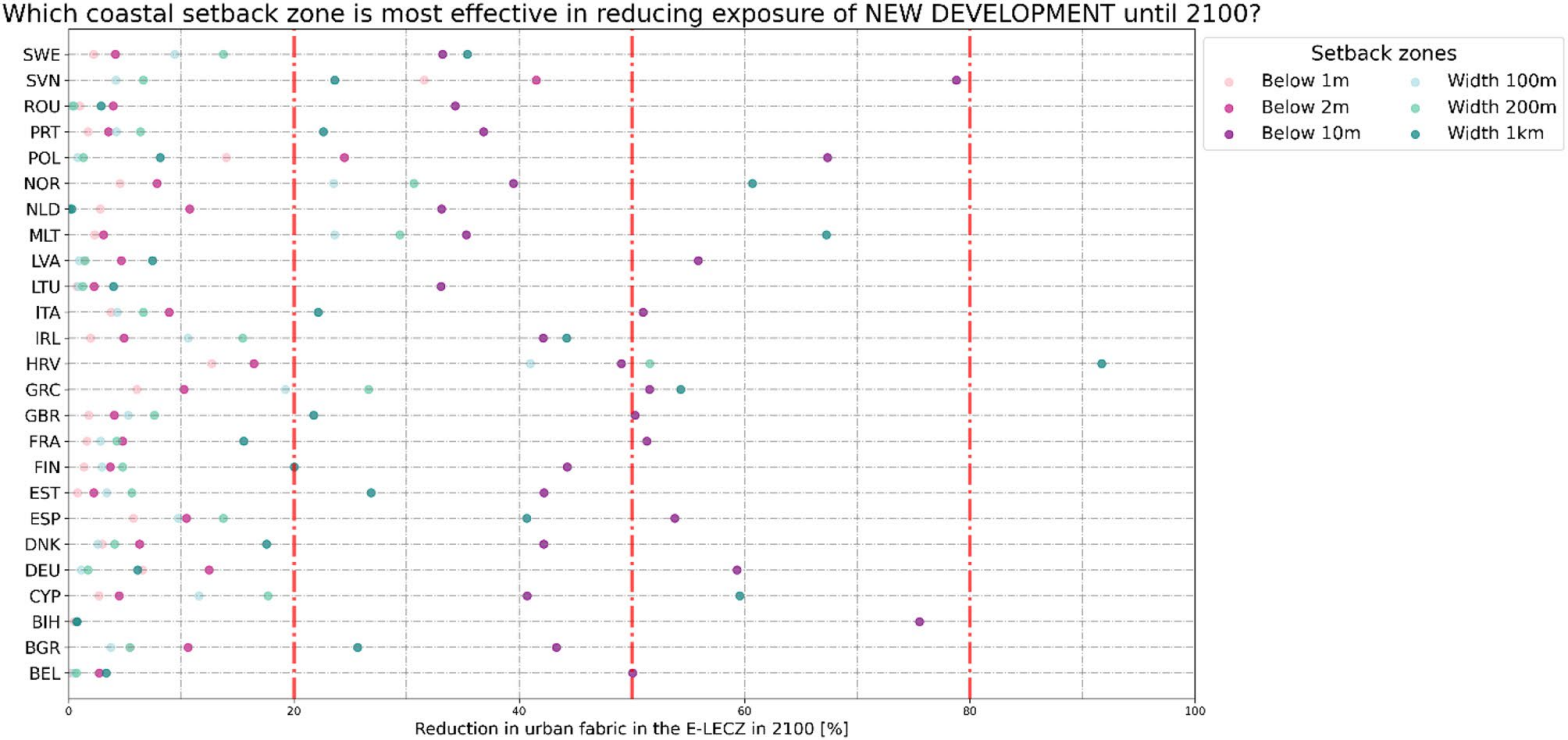
Reduction in exposure of new urban fabric in the E-LECZ under different setback zones until 2100 [high urban growth scenario].

For two-third of the countries, the implementation of coastal setback zones that restrict further development below 10 m of elevation is most effective in reducing future urban exposure (see Figs. 6 and 7). The reduction in overall exposure of urban areas in 2100 could be up to 35% in Bosnia-Herzegovina, followed by Great Britain and France, which both have a reduction potential of around 28%. However, in Croatia, Cyprus, Norway, Greece, Malta, and Sweden, a 1 km wide setback zone leads to the largest reduction in urban exposure. All these countries have in common that large parts of the coast have a relatively steep coastal profile (often rocky, with pocket beaches). Thus, the coastal floodplain is narrow and the area below 10 m is often smaller than the 1 km inland buffer, leading to a greater urban area not being exposed in the future.

Although, the overall reduction potential of setback zones may seem small at first, this changes when we look at the reduction potential of new urban development only. Figure 7 shows how high the potential of setback zones is in reducing the exposure of new urban areas in individual countries. In addition, we have highlighted in Fig. 7 which measures would lead to a 20%, 50%, and 80% reduction in urban exposure of new development in 2100. In 15 of the 25 countries studied, a setback zone of either a distance of 100 m or 1 km; or 10 m elevation results in a reduction of exposure of at least 50% in 2100 under a high urban growth scenario. Overall, future urban exposure in the EU can be reduced by 47% in the E-LECZ if the most effective setback zone for each country is implemented (considering a high urban growth scenario).

Interestingly, the most effective coastal setback zone does not depend on the urban growth scenario in any country (see Supplementary Fig. 1). This indicates that the morphology of the coastal floodplain could be the main factor influencing how well setback zones work to reduce urban exposure. Therefore, the best setback zone per country remains constant throughout all urban growth scenarios, which highlights that the uncertainty in urban growth is not a defining factor when designing coastal setback zones within a specific country. However, the demand for urban fabric in the future or the development under a specific urban growth scenario has a significant influence on the overall urban exposure in flood-prone regions.

### Retreat prospects

**Figure 8:**
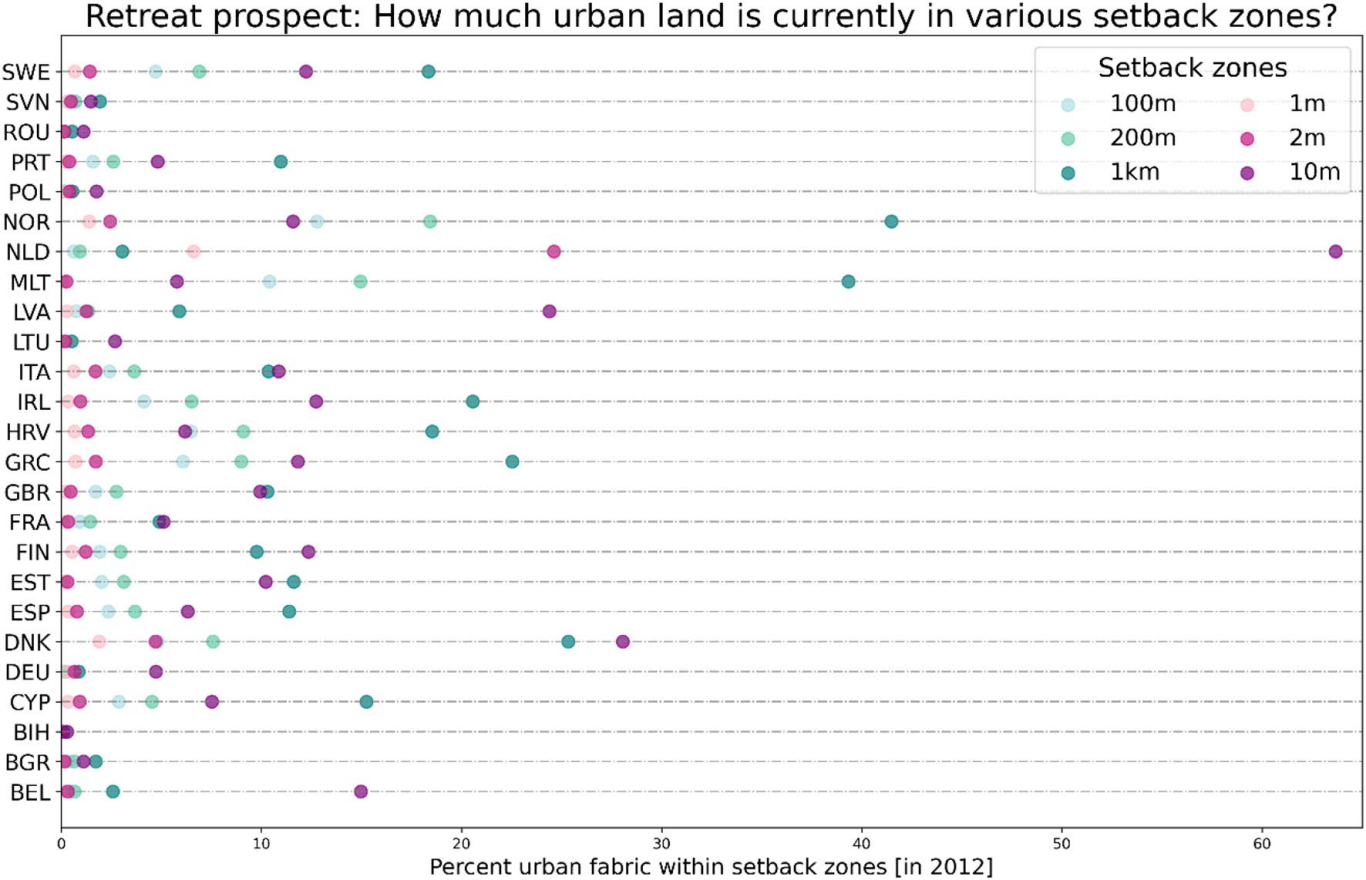
Retreat prospect of EU member states (plus NOR, BIH, GBR): the extent of urban areas that are currently located in different setback zones [using CORINE 2012 data].

Figure 8 shows the share of the urban areas in the different setback zones to each nation’s overall urban areas in 2012 (all numbers can be found in Supplementary Table 1) to illustrate and quantify possible retreat prospects for every EU coastal member state. Relocating all urban areas away from high-risk areas would necessitate the relocation of up to 64% of all urban areas within a country (see Fig. 8, Netherlands). Around 64% of all urban areas in the Netherlands are currently below 10 m. Interestingly, in Norway, a high proportion of urban areas are located near the coast (more than 40% within 1 km), but only a quarter of these are low-lying. In contrast, in Denmark, the proportion of urban areas within 1 km and below 10 m of elevation is similar, which may be related to the geomorphology, as Denmark is generally low-lying, unlike Norway, where the coastal profile is often steep. In terms of total numbers, Great Britain has the largest extent of urban areas (2171 km^2^), followed by Italy (1708 km^2^), in the first kilometer from the coast, even though this represents only 10% of the total extent of urban areas of both countries. Moreover, Great Britain (2093 km^2^) and Italy (1790 km^2^) are second and third in terms of the total extent of urban areas in the LECZ (below 10 m elevation), illustrating that retreat would be challenging for both countries. Overall, as Fig. 8 shows, in 13 out of 25 countries, the highest amount of urban area is found in the 1 km wide coastal strip, while in the other countries (12), the highest amount of urban area is below 10 m elevation.

## Discussion

### Future urban extent projections

Numerous studies of European cities have demonstrated that the urban extent increased even when population decreased^27,28^. Our constructed spatially explicit urban extent projections also show the same pattern, as urban extent grows over time under all scenarios, albeit more slowly in scenarios with declining populations. The urban extent projections show how urbanization and GDP growth in high-income nations are related. In contrast, urban development in lower-income nations is more influenced by population growth as we approach the end of the century. Other studies have also noted this trend (see, for instance, Seto et al. 2011's^29^ meta-analysis of worldwide urban land expansion). According to Gao and O'Neil^30^, population and built-up land change differently over time: typically, in observational data, population can increase or decrease, while built-up land either expands or remains the same. This relates to the fact that even after being repurposed, developed land is frequently not converted back to undeveloped. Furthermore, projections of population dynamics for the future show no indication that the world's trend toward urbanization will slow down or reverse^24,31^.

### Setback potential: the effect of setback zones on reducing future urban exposure

This study represents a first-order EU-wide assessment of the efficacy of various coastal setback zones as an adaptation measure in reducing future urban exposure. Our primary finding is that by incorporating setback zones, the effects of sea level rise on future urban exposure can be potentially mitigated. Our results suggest that establishing setback zones appears especially beneficial for nations with a long coastline such as Italy, Norway, France, Sweden, or Great Britain, as it is inherently more expensive and challenging to adapt to and protect long coastal stripes^32^. However, the most effective type of setback zone in reducing future urban exposure, depends primarily on the type of coast and the morphology of the coastal profile/floodplain, and thus is country or location specific. Additionally, results show that a coastal setback zone by a certain elevation, i.e., development is restricted to the area above a certain elevation, would be one of the most effective ways for most EU countries to reduce urban exposure in the E-LECZ. To our knowledge, this type of setback zone is currently not implemented in EU member states, but results highlight that the implementation can offer large benefits in the future. Therefore, we propose defining setback zones by elevation as an additional criterion for coastal planning in EU nations. The application of a fixed setback distance has the advantage of simplicity but presents challenges when applied to a wide range of geomorphology, values, and cultural contexts. Our findings imply that a greater level of intervention (setbacks with a higher distance or elevation threshold) would be required if coastal managers are interested in lowering exposure in the E-LECZ.

Setback zones are a low-regret strategy as the direct cost of implementing them is relatively low^15,33^.This however does not account for potential opportunity costs that may arise, particularly for activities and social groups for which proximity to the coast is vital. Such examples may include, among others, land development, tourism infrastructure and activities or fisheries. These costs are not distributed uniformly, neither spatially^34^ nor socially and distributional effects that may arise^35^ would need to be considered when planning setbacks. Nevertheless, opportunity costs are more often associated with unmanaged retreat rather than with planned interventions (e.g. setback zones), which can result in long-term benefits^36^. Further, setback zones could not only decrease coastal urban exposure but also increase the available space for nature-based adaptation^37^, thus broadening the options for future adaptation and helping to avoid lock-ins or path dependence, where continuous upgrading of protection measures is required as sea levels rise. It is evident that if we want to continue building directly on the coast and hold the current coastline/defense line, it will become very expensive in the future^38^. Our study shows that refraining from settling in high-risk areas can reduce future urban exposure to coastal hazards in the EU by 46% until 2100 (considering only new urban development under a high urban growth scenario and the most effective setback zone for every country). Although such solutions often face opposition from local communities and stakeholders due to the redistribution of benefits^39,40^, they are slowly being implemented or at least discussed at local scales^41^ as awareness of rising sea levels increases and a shift in policy priorities towards optimizing and re-evaluating the effectiveness of expensive engineered adaptation solutions occurs^39^. Therefore, countries possibly have different attitudes towards transformative land use planning. Countries with a long history in coastal protection and erosion may be more receptive to discussing landuse planning solutions (e.g. setback zones, retreat, managed realignment), at least where population densities are low (e.g. in parts of the UK or German Baltic Sea coast^41,42^). On the other hand, nations are likely to struggle in implementing setbacks, if development has historically taken place directly at the coast, reinforced by coastal tourism (e.g. in the Mediterranean); or where an over-reliance on protective technology exists; and there is a low tolerance for disasters (NW Europe) or limited space. Some studies have demonstrated that a more sustainable approach to managing coastal risks would be to focus on densifying safe areas rather than accelerating urban growth and land take-up in the coastal lowlands, because the way we plan, design, and develop urban settlements will determine future patterns of exposure, vulnerability, and resilience to sea-level rise^43^.

### Barriers and limitations to implementing coastal setback zones

Designing and implementing coastal setback zones and setback policies to reduce coastal erosion and coastal flooding is a difficult task that must take into account (1) coastal dynamics, such as sea-level rise and changing storms; (2) country-specific needs like the preservation of natural and cultural landscapes^12^ and (3) regional specific characteristics like the geomorphology of the coastal floodplain.

Public opposition is one of the biggest barriers to the implementation of setbacks. This is especially likely if the public thinks setbacks are too large or if, landowners lose the right to build on their own land, which puts them at a significant disadvantage^33^ and may lead to an unequitable distribution of benefits^35^. Further, pressure to develop the coastal zone is present in many coastal areas, particularly when tourism development is being promoted. As a result, coastal regulations frequently fail to prevent development within the setback zone. In addition, proposing a large coastal setback zone is only feasible for countries that have a large proportion of land outside the floodplain. A setback zone with an elevation threshold of 10 m or a distance of 1 km may therefore not be feasible on small island states like Malta or in nations where the proportion of the coastal floodplain is very high, such as in the Netherlands. However, if the setback zone is too small, its size will need to be periodically evaluated and adjusted due to the fact that sea-level rise will reduce the size of the setback zones in the future. Furthermore, a challenge in implementing a setback zone is the definition of the baseline^4^used to determine the landward or seaward boundary depending on; legal definitions; local conditions of the coastline, water levels and tides; or data availability constraints.

### Retreat prospects for European countries

Retreat is currently discussed as a risk reduction option by international organizations such as the United Nations (e.g. in the Sendai Framework for Disaster Risk Reduction), which might have a significant impact on facilitating large-scale planning^44^. Managed retreat has its own set of political, social and legal difficulties making it neither a low-regret decision nor one that can be easily undone. However, it may have a better benefit–cost ratio than protection via dikes^42^, especially in areas with low population densities^15,42^. The few retreat projects that exist within Europe have mainly been implemented to restore salt marshes and support coastal defence systems in more rural areas. Examples can be found in Germany (Langeoog Island^45^, Geltinger Birk^41^) or the UK (e.g. Freiston Shore^46^). However, in the Unites States, which has the longest history of retreat programs globally^47^, retreat has been documented primarily in urban areas^48,49^. The U.S. government has mostly deployed the voluntary buyout methods of flood-prone properties to facilitate retreat^50^. This raises the question of how retreat projects in urban settings could potentially unfold in Europe if similar policy incentives are implemented. We believe that any integrated regional planning strategy focused on combating sea-level rise and climate-related hazards should include the practice of relocating or resettling people and activities from hazardous locations to safe ones^51^ because holding the line for the entire European coastline is not affordable. Therefore, a reorientation in coastal management in Europe will be required to invent, implement, and improve socially acceptable and equitable retreat options. A strategic, managed retreat in Europe will require a significant amount of innovation and labor—in both research and practice—to make it an effective and equitable adaptation option.

To obtain a first idea of the retreat prospect in the EU coastal member states, we calculated the percentage of the urban area that is currently (in 2012) located in different setback zones. This provides a first-order assessment of the upfront costs/resources that would be needed for a complete coastal retreat in every EU country. The rewards for achieving this task can be very high, as coastal climate-related hazards are increasing and people will continue to leave dangerous areas as climate hazards increase, whether prompted by disasters, market forces, or government intervention^44^. We believe that if the urban extent within a country for a specific setback zone is high it will be more unlikely that countries/communities implement such retreat policies.

However, if perceived risk levels are increasing, retreat or setback zones may become more acceptable in the future. Future research should therefore examine how attitudes toward setback zones and managed retreat change as the risk of climate-related hazards rises. This information could help determine whether setback zones and managed retreat are likely to present an opportunity in high-risk areas of EU coastal member states, or whether engineering interventions are required or constitute the preferred solution. Retreat requires a decadal lead time to be planned and implemented equitably. Furthermore, many decisions taken today have a long legacy effect and create path dependencies, closing off some options in the future^52^. Therefore, retreat is best employed as one of several tactics in the pursuit of a coastal management strategy with objectives ranging from environmental preservation to economic development.

### Limitations of the study

**Figure 9:**
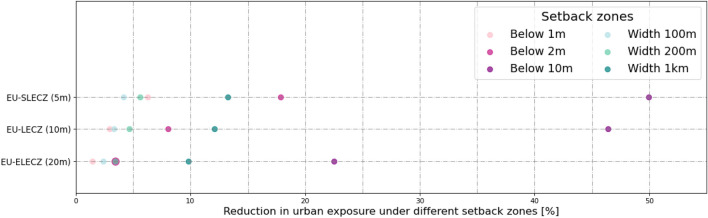
Sensitivity analysis of different coastal definitions on the results for Europe using three different coastal floodplain definitions (hydrologically connected to the sea; below 20 m (ELECZ), 10 m (LECZ), and 5 m (SLECZ)).

The scope of the study is to provide a first-order assessment and indication of the avoided potential urban exposure for various types of setback zones, and thus simplifications in the representation of the underlying processes are inherent. The limitations of our study include that (1) we only account for horizontal and not vertical urban growth, which could lead to more sustainable urban development from a land-uptake perspective. The lack of European-wide urban height datasets is one barrier at the moment. Another restriction (2) is that Corine 2000 and Corine 2012 have some differences in their coastlines (e.g. in harbors). This has an impact on the study's coastal setback zone designs, particularly for the 100 m, 200 m, and 1 km distance setback zones. However, the overall trends and results of the study will be unaffected by this. The study's urban exposure results vary depending on how (3) the coastal floodplain is defined (in this study, we used the E-LECZ). To better quantify the impact of different coastal definitions on the results for Europe, we analyzed the sensitivity using three different coastal floodplains with different elevation thresholds (see Fig. 9). It is evident that the smaller the coastal floodplain, the greater the relative reduction in urban exposure from elevation-specific setbacks, indicating that the results of the study tend to be conservative/at the lower bound (Fig. 9).

## Methods and data

### Urban change modelling approach

**Figure 10:**
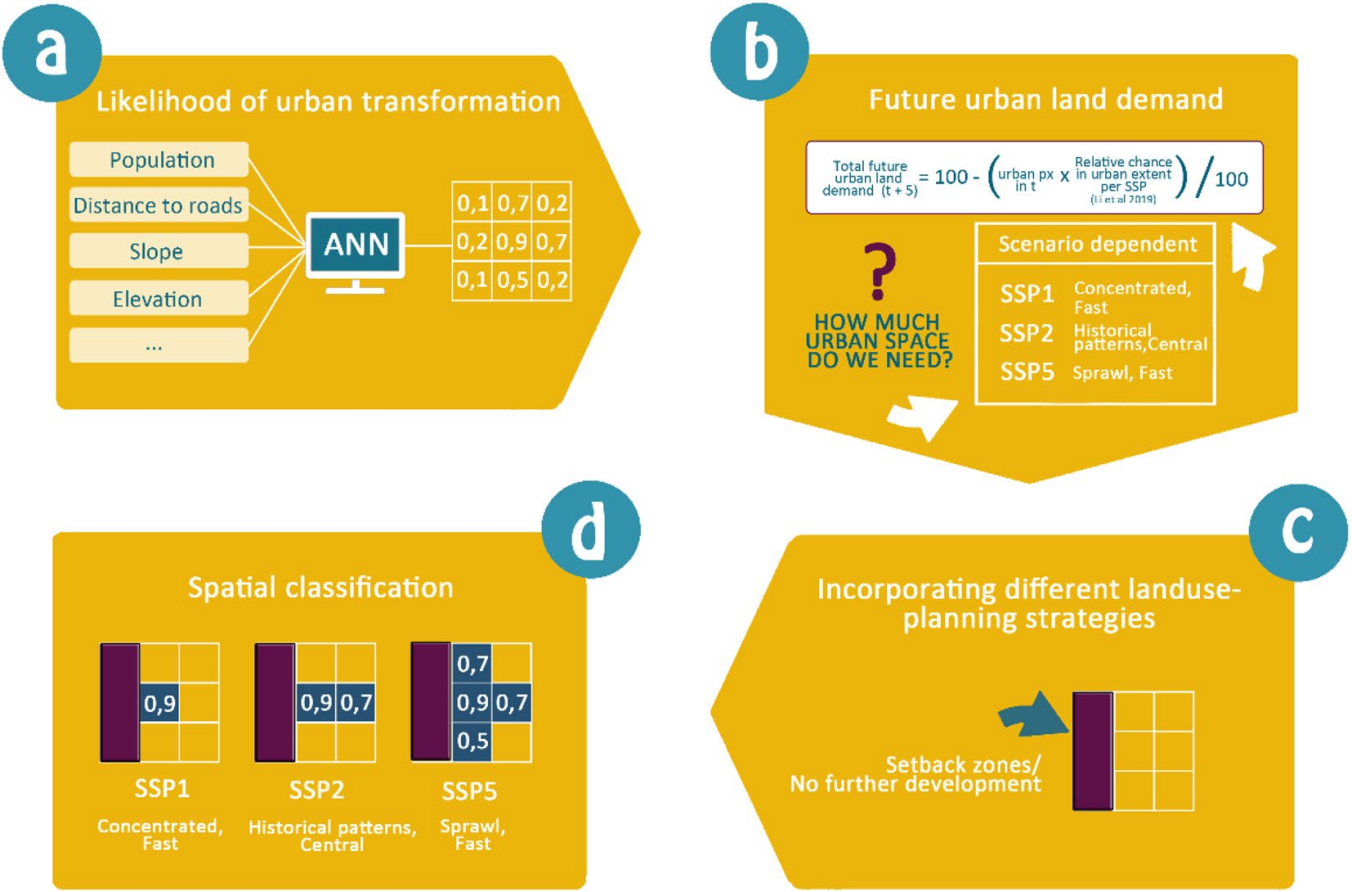
Urban change modelling chain. Step (**a**) visualizes the use of the MLP ANN to develop the urban development potential/likelihood surface; Step (**b**) shows the calculation of the future demand for urban land; Step (**c**) illustrates the incorporation of different setback zone scenarios, and in Step (**d**) the final spatial reclassification to generate the spatially-explicit projections is shown.

In this study, we extend the urban change model, developed by Wolff et al.^18^, to account for restricted urban development zones, called setback zones (see Fig. 10). We develop country-specific urban change models for all coastal EU Member States (plus BIH, GBR, NOR) for which data and SSP assumptions were available.

### Urban development potential/likelihood

We use a Multi-Layer-Perceptron Artificial Neural Network (MLP ANN) to estimate the urban development potential for every coastal EU member state plus BIH, GBR, and NOR, at a 100 m resolution. For each country, the MLP ANN was trained and tested using input data from 2000 to predict urban land cover in 2012 (see Table 2). We define urban areas as locations dominated by artificial surfaces, which corresponds to the artificial surface class (code 1) from CORINE that includes residential, industrial, commercial, transport, and green urban areas. We chose nine variables that were frequently described in the literature as driving urban development and for which European-level data was available. These variables are used as input layers in the MLP.

**Table 2:** Summary of data used for input and output variables of the MLP.

Variables		Source	Spatial resolution	Pre-processing notes
Input	Distance to forest	CORINE 2000	100 m	Corine classes used: 311, 312, 313, Euclidean distance to forest 2000
	Distance to grassland	CORINE 2000	100 m	Corine classes used: 321, 322, 323, 324, Euclidean distance to grassland 2000
	Distance to urban	CORINE 2000	100 m	Corine classes used: 111, 112, 121, 122, 123, 124, 131, 132, 133, 141, 142, Euclidean distance to urban 2000
	Distance to arable land	CORINE 2000	100 m	Corine classes used: 211, 212, 213, 221, 222, 223, 231, 241, 242, 243, 244, Euclidean distance to arable land 2000
	Distance to roads	OSM	100 m	Main roads (fclass: primary, motorway, secondary), Euclidean distance to roads
	Distance to coast	Based on CORINE coastline	100 m	Euclidean distance to coastline
	Population density	GPW v4 2000	1 km	Total population in 2000 per km^2^, resampled to 100 m
	Elevation	MERIT DEM	90 m	Elevation in m, resampled to 100 m
	Slope	Derived from MERIT DEM	90 m	Percent increase in slope, resampled to 100 m
	Setback zones	Based on CORINE coastline/ MERIT DEM	100 m/ 90 m	Distance to coast increments, Setback zones: 100 m, 200 m, 300 m, 500 m, 1 km; Hydrological connected elevation increments, Setback zones: 0.5 m, 1 m, 1.5 m, 2 m, 2.5 m, 3 m, 5 m, 10 m; resampled to 100 m
Output	Urban	CORINE 2012	100 m	Reclassification of Corine data to Urban = 1, Rural = 0; Corine classes used: 111, 112, 121, 122, 123, 124, 131, 132, 133, 141, 142

The output layer consists of a single variable that indicates the probability or likelihood that a pixel will become urban in the next time step. To determine the best MLP architecture for each country (i.e., the number of neurons in each layer, the connection patterns between layers, the activation function, and the learning methods), we performed a sensitivity analysis with five to six different network architectures. Table 3 shows a summary of the best model architecture per country and Table 4 the performance measures for every country.

**Table 3:** Best performing MLP model architecture per country.

Country	Hidden layer 1 [Nr of neurons]	Hidden layer 2 [Nr of neurons]	Hidden layer 3 [Nr of neurons]	Hidden layer 4 [Nr of neurons]	Activation function for the hidden layer	Under sampling factor	Activation function output layer	Optimization algorithm
BEL	400	400	400	200	Relu	2	Sigmoid	Adam
BGR	400	400	400	200	Relu	8	Sigmoid	Adam
BIH	400	400	400	200	Relu	12	Sigmoid	Adam
CYP	600	600	600	300	Relu	5	Sigmoid	Adam
DEU	1000	1000	800	400	Relu	False	Sigmoid	Adam
DNK	400	400	400	200	Relu	10	Sigmoid	Adam
ESP	400	400	400	200	Relu	5	Sigmoid	Adam
EST	400	400	400	200	Relu	12	Sigmoid	Adam
FIN	600	600	600	400	Relu	12	Sigmoid	Adam
FRA	600	600	600	300	Relu	10	Sigmoid	Adam
GBR	600	600	300	–	Relu	10	Sigmoid	Adam
GRC	800	800	300	–	Relu	10	Sigmoid	Adam
HRV	400	400	400	200	Relu	8	Sigmoid	Adam
IRL	600	600	300	–	Relu	10	Sigmoid	Adam
ITA	600	600	300	–	Relu	12	Sigmoid	Adam
LTU	600	600	400	150	Relu	12	Sigmoid	Adam
LVA	600	600	600	400	Relu	10	Sigmoid	Adam
MLT	400	400	400	200	Relu	False	Sigmoid	Adam
NLD	800	800	800	400	Relu	5	Sigmoid	Adam
NOR	400	400	400	200	Relu	10	Sigmoid	Adam
POL	400	400	400	200	Relu	10	Sigmoid	Adam
PRT	600	600	600	400	Relu	5	Sigmoid	Adam
ROU	400	400	200	–	Relu	8	Sigmoid	Adam
SVN	400	400	200	–	Relu	8	Sigmoid	Adam
SWE	400	400	200		Relu	8	Sigmoid	Adam

**Table 4:** Performance measures of the MLP for predicting urban extent in 2012 for every country.

Country	Overall accuracy	Precision	Recall	Negative predictive value	Specificity	F1-Score
BEL	0.994	0.992	0.982	0.995	0.998	0.987
BGR	0.990	0.877	0.913	0.996	0.994	0.895
BIH	0.995	0.874	0.843	0.997	0.998	0.858
CYP	0.989	0.939	0.941	0.994	0.994	0.940
DEU	0.963	0.839	0.763	0.975	0.985	0.799
DNK	0.991	0.959	0.936	0.994	0.996	0.948
ESP	0.985	0.737	0.624	0.990	0.994	0.676
EST	0.997	0.939	0.942	0.999	0.999	0.940
FIN	0.995	0.823	0.833	0.998	0.997	0.828
FRA	0.987	0.940	0.832	0.989	0.997	0.883
GBR	0.977	0.902	0.825	0.984	0.992	0.862
GRC	0.989	0.856	0.795	0.993	0.996	0.825
HRV	0.993	0.924	0.871	0.995	0.997	0.897
IRL	0.993	0.860	0.847	0.996	0.997	0.854
ITA	0.990	0.951	0.857	0.992	0.997	0.902
LTU	0.992	0.880	0.892	0.996	0.996	0.886
LVA	0.993	0.842	0.815	0.996	0.997	0.828
MLT	0.996	0.994	0.992	0.997	0.998	0.993
NLD	0.981	0.954	0.926	0.986	0.991	0.940
NOR	0.999	0.970	0.960	1.000	1.000	0.965
POL	0.973	0.868	0.662	0.978	0.993	0.751
PRT	0.991	0.920	0.882	0.995	0.996	0.901
ROU	0.979	0.797	0.825	0.990	0.988	0.811
SVN	0.991	0.886	0.859	0.995	0.996	0.872
SWE	0.998	0.950	0.908	0.999	0.999	0.928

### Future demand for urban land

Following the methods described in Wolff et al.^18^, we used the relative changes of the non-spatial urban extent growth model of Li et al.^17^ for every country between 2020 and 2100. The growth model of Li et al.^17^ was developed using a 22-year time series of urban extent from 1992 to 2013 derived from satellite observations as well as historical indicators of population and GDP values. According to Li et al.^17^, the mean accuracy of the 1 km urban extent data for Europe is 95% compared to fine-resolution land cover data. The developed country-specific models were then used to project future urban growth under the Shared Socioeconomic Pathways (SSP) to the end of the twenty-first century, using future population and GDP data from the SSP database. We used these relative non-spatial urban growth values in order to calculate the total number of future urban grid cells for each country, every SSP and time step (see Fig. 10).

### Setback zone scenarios

In order to account for coastal setback zones in the EU, we developed different spatial setback zone scenarios and integrated them into the urban change model of Wolff et al.^18^. For this purpose, we developed two different forms of setback zones. The first form is width-dependent and allows no further development within a specific distance from the coast (100 m, 200 m, 1 km), similar to, for instance, Article 8-2 of the Mediterranean ICZM Protocol, which prescribes the establishment of a 100-m zone^53^, where new construction is not allowed. Further, we looked into the literature^12,14,54,55^ and conducted a short questionnaire survey (which is available at https://www.umfragen.uni-kiel.de/index.php/935635?lang=de) to which five coastal experts from Italy, Germany, Croatia, France and Great-Britain responded, supporting the selection of setback zone width.

The second form is dependent on the elevation, as low-lying coastal floodplains are potentially the most exposed to climate-related hazards. Therefore, we developed coastal floodplains below different elevation thresholds, specifically 1 m, 2 m, and 10 m, that are hydrologically connected to the ocean based on the MERIT DEM^56^ and which cover the historical and projected extreme sea levels within Europe^57^.

### Spatial reclassification

The last step in the modelling chain is the reclassification of the urban development potential grid using the future urban land demand calculations. The highest likelihoods of urban development were converted into urban, based on the total number of future urban grid cells required in each time step. We used CORINE 2012 as a base year to create spatially explicit urban extent scenarios that incorporate different setback zones, SSPs, and time steps until 2100. We produced various scenario combinations for the coastal European Member States plus NOR, GBR, and BIH where the global SSP assumptions and data were available.

### Future coastal exposure calculation

To analyze the extent of urban space potentially exposed to climate-related hazards such as coastal flooding or SLR, we calculated the extended low elevation coastal zone (E-LECZ), which represents the area below 20 m elevation that is hydrologically connected to the ocean. The E-LECZ was then overlaid with the various setback zone scenarios to see how the potentially exposed area changes when different setback zones are implemented.

### High and low urban extent layers

The SSP scenario that leads to the highest or lowest urban development across Europe varies. For instance, while SSP5 produces the highest urban extent in France, Italy or Poland, SSP3 results in the highest urban extent in Latvia, Bosnia and Herzegovina or Lithuania (for a table showing the highest and lowest SSP scenarios for all countries, we refer readers to Table 1 in the result section). To minimize the different scenario combinations for the analysis but still cover the full range of uncertainty, we decided to develop one high and one low urban extent scenario for each setback zone scenario. Thus, the high urban extent scenario reflects the SSP that produces the highest urban extent for every country and vice versa for the low urban extent scenario.

## Supplementary Information

Supplementary Information.

## Data Availability

All data that support the findings of this study are included within the article (and any supplementary files). The EU urban extent projections for 2100 under different setback zones and a high and low urban growth with a 100 m resolution are available at https://figshare.com/s/04da78fc90bd85fcedfe and the projections without a setback zone are available here https://figshare.com/s/797bc6336171abb65bb0.

## Code availability

The code for the urban model is available at: https://figshare.com/s/36f9c0a32ac3ba5945a7.

## References

[1] Wing OEJ (2022). Inequitable patterns of US flood risk in the Anthropocene. Nat. Clim. Chang..

[2] Fox-Kemper, B. *et al.* Ocean, cryosphere and sea level change. In *Climate Change 2021: The Physical Science Basis. Contribution of Working Group I to the Sixth Assessment Report of the Intergovernmental Panel on Climate Change* (eds Masson-Delmotte, V. *et al*.) 1211–1362 (Cambridge University Press, 2021). 10.1017/9781009157896.011.

[3] Vousdoukas MI (2020). Economic motivation for raising coastal flood defenses in Europe. Nat. Commun..

[4] Neumann B, Vafeidis AT, Zimmermann J, Nicholls RJ (2015). Future coastal population growth and exposure to sea-level rise and coastal flooding—a global assessment. PLoS ONE.

[5] Andreadis KM (2022). Urbanizing the floodplain: Global changes of imperviousness in flood-prone areas. Environ. Res. Lett..

[6] Oppenheimer M (2019). Sea level rise and implications for low-lying islands. Coasts Commun..

[7] Li M, Verburg PH, van Vliet J (2022). Global trends and local variations in land take per person. Landsc. Urban Plan..

[8] Gao J, O’Neill BC (2020). Mapping global urban land for the 21st century with data-driven simulations and Shared Socioeconomic Pathways. Nat. Commun..

[9] IPCC. Summary for Policymakers. In *Climate Change 2022: Impacts, Adaptation, and Vulnerability. Contribution of Working Group II to the Sixth Assessment Report of the Intergovernmental Panel on Climate Change* In Press (2022).

[10] Kirby JA, Masselink G, Essex S, Poate T, Scott T (2021). Coastal adaptation to climate change through zonation: A review of coastal change management areas (CCMAs) in England. Ocean Coast. Manag..

[11] Mechler R, Schinko T (2016). Identifying the policy space for climate loss and damage. Science.

[12] Sanò M (2011). The role of coastal setbacks in the context of coastal erosion and climate change. Ocean Coast. Manag..

[13] UNEP/MAP/PAP. In *Protocol on Integrated Coastal Zone Management in The Mediterranean* (2008).

[14] Rochette, J., du Puy-Montbrun, G., Wemaëre, M. & Billé, R. In *Coastal setback zones in the Mediterranean: A study on Article 8-2 of the Mediterranean ICZM Protocol* (2010).

[15] Lincke D (2020). The effectiveness of setback zones for adapting to sea-level rise in Croatia. Reg. Environ. Change.

[16] Haasnoot M, Lawrence J, Magnan AK (2021). Pathways to coastal retreat. Science.

[17] Li X, Zhou Y, Eom J, Yu S, Asrar GR (2019). Projecting global urban area growth through 2100 based on historical time series data and future shared socioeconomic pathways. Earth’s Future.

[18] Wolff C, Nikoletopoulos T, Hinkel J, Vafeidis AT (2020). Future urban development exacerbates coastal exposure in the Mediterranean. Sci. Rep..

[19] O’Neill BC (2014). A new scenario framework for climate change research: The concept of shared socioeconomic pathways. Clim. Change.

[20] Gesch DB (2018). Best practices for elevation-based assessments of sea-level rise and coastal flooding exposure. Front. Earth Sci..

[21] Hooijer A, Vernimmen R (2021). Global LiDAR land elevation data reveal greatest sea-level rise vulnerability in the tropics. Nat. Commun..

[22] O’Neill BC (2017). The roads ahead: Narratives for shared socioeconomic pathways describing world futures in the 21st century. Glob. Environ Change.

[23] Samir KC, Lutz W (2014). The human core of the shared socioeconomic pathways: Population scenarios by age, sex and level of education for all countries to 2100. Glob. Environ. Change.

[24] Jiang L, O’Neill BC (2017). Global urbanization projections for the shared socioeconomic pathways. Glob. Environ. Chang..

[25] Jones B, Oeill BC (2016). Spatially explicit global population scenarios consistent with the Shared Socioeconomic Pathways. Environ. Res. Lett..

[26] Kc S, Lutz W (2017). The human core of the shared socioeconomic pathways: Population scenarios by age, sex and level of education for all countries to 2100. Glob. Environ. Chang..

[27] Barranco RR, Silva FBE, Marin-Herrera M, Lavalle C (2014). Integrating the MOLAND and the urban Atlas Geo-databases to analyze urban growth in European cities. J. Map. Geogr. Libr..

[28] Kasanko M (2006). Are European cities becoming dispersed?. Landsc. Urban Plan..

[29] Seto KC (2011). Exploring the dynamics of migration to mega-delta cities in Asia and Africa: Contemporary drivers and future scenarios. Glob. Environ. Change.

[30] Gao J, O’Neill B (2021). Different spatiotemporal patterns in global human population and built-up land. Earth’s Future.

[31] United Nations, Department of Economic and Social Affairs, Population Division. World Population Prospects 2019: Highlights (ST/ESA/SER.A/423). https://population.un.org/wpp/publications/files/wpp2019_highlights.pdf (2019).

[32] McEvoy S, Haasnoot M, Biesbroek R (2021). How are European countries planning for sea level rise?. Ocean Coast. Manag..

[33] Linham, M. M. & Nicholls, R. J. *Technologies for climate change adaptation: Coastal erosion and flooding*. (UNEP Riso Centre on Energy, Climate and Sustainable Development, 2010).

[34] Kousky C, Walls M (2014). Floodplain conservation as a flood mitigation strategy: Examining costs and benefits. Ecol. Econ..

[35] Bisaro A (2019). Coastal adaptation through urban land reclamation: Exploring the distributional effects. Erde.

[36] Hanna, C., White, I. & Glavovic, B. Managed retreat in practice: Mechanisms and challenges for implementation. In *Oxford Research Encyclopedia of Natural Hazard Science* (Oxford University Press, 2019). 10.1093/acrefore/9780199389407.013.350.

[37] Schuerch M (2019). Author correction: Future response of global coastal wetlands to sea-level rise. Nature.

[38] Lincke D, Hinkel J (2018). Economically robust protection against 21st century sea-level rise. Glob. Environ. Chang..

[39] de la Vega-Leinert AC, Stoll-Kleemann S, Wegener E (2018). Managed realignment (MR) along the Eastern German Baltic sea: A catalyst for conflict or for a coastal zone management consensus. J. Coast. Res..

[40] Groen L, Alexander M, King JP, Jager NW, Huitema D (2022). Re-examining policy stability in climate adaptation through a lock-in perspective. J. Eur. Public Policy.

[41] Schernewski G, Bartel C, Kobarg N, Karnauskaite D (2018). Retrospective assessment of a managed coastal realignment and lagoon restoration measure: The Geltinger Birk, Germany. J. Coast Conserv..

[42] Lincke D, Hinkel J (2021). Coastal migration due to 21st century sea-level rise. Earth’s Future.

[43] Dodman, D. M. *et al. Cities, settlements and key infrastructure*. In *Climate Change 2022: Impacts, Adaptation, and Vulnerability. Contribution of Working Group II to the Sixth Assessment Report of the Intergovernmental Panel on Climate Change*. https://eprints.whiterose.ac.uk/185076/. (2022).

[44] Siders AR, Hino M, Mach KJ (2019). The case for strategic and managed climate retreat. Science.

[45] Hofstede JLA (2019). On the feasibility of managed retreat in the Wadden Sea of Schleswig-Holstein. J. Coast Conserv..

[46] Kiesel J, Schuerch M, Möller I, Spencer T, Vafeidis A (2019). Attenuation of high water levels over restored saltmarshes can be limited. Insights from Freiston Shore, Lincolnshire, UK. Ecol. Eng..

[47] Hino M, Field CB, Mach KJ (2017). Managed retreat as a response to natural hazard risk. Nature Clim. Change.

[48] Binder SB, Greer A (2016). The devil is in the details: Linking home buyout policy, practice, and experience after hurricane sandy. PaG.

[49] Mach KJ (2019). Managed retreat through voluntary buyouts of flood-prone properties. Sci. Adv..

[50] Zavar E, Hagelman RR (2016). Land use change on US floodplain buyout sites, 1990–2000. Disaster Prevent. Manage..

[51] Dachary-Bernard J, Rey-Valette H, Rulleau B (2019). Preferences among coastal and inland residents relating to managed retreat: Influence of risk perception in acceptability of relocation strategies. J. Environ. Manage..

[52] Haasnoot M (2021). Long-term sea-level rise necessitates a commitment to adaptation: A first order assessment. Clim. Risk Manag..

[53] United Nations Environment Programme. In *Protocol on Integrated Coastal Zone Management in the Mediterranean*. (United Nation Environment Programme, 2008).

[54] Sas E, Fischhendler I, Portman ME (2010). The demarcation of arbitrary boundaries for coastal zone management: The Israeli case. J. Environ. Manage..

[55] EUCC. *Policy Instruments for ICZM in Nine Selected European Countries*. https://eucc-d-inline.databases.eucc-d.de/files/documents/00000621_EUCC_ICZM_policy.pdf (2000).

[56] Yamazaki D (2017). A high-accuracy map of global terrain elevations: Accurate Global Terrain Elevation map. Geophys. Res. Lett..

[57] Kirezci E (2020). Projections of global-scale extreme sea levels and resulting episodic coastal flooding over the 21st Century. Sci. Rep..

