# Supplementary material for: Setback zones can effectively reduce exposure to sea-level rise in Europe
Source: Sci Rep. 2023 Apr 4;13:5515. doi: 10.1038/s41598-023-32059-9 (PMC10073184; doi:10.1038/s41598-023-32059-9)

Supplementary Material

Setback zones can effectively reduce exposure to sea-level rise in Europe

Claudia Wolff[1*], Hedda Bonatz[1], Athanasios T. Vafeidis[1]

1Coastal Risks and Sea-Level Rise Research Group, Department of Geography, Christian-Albrechts University Kiel, Kiel, Germany

*Corresponding Author: e-mail: wolff@geographie.uni-kiel.de, tel: +49-431-880 5319

Supplementary Figure 1: Urban exposure reduction under a high and low urban growth scenario

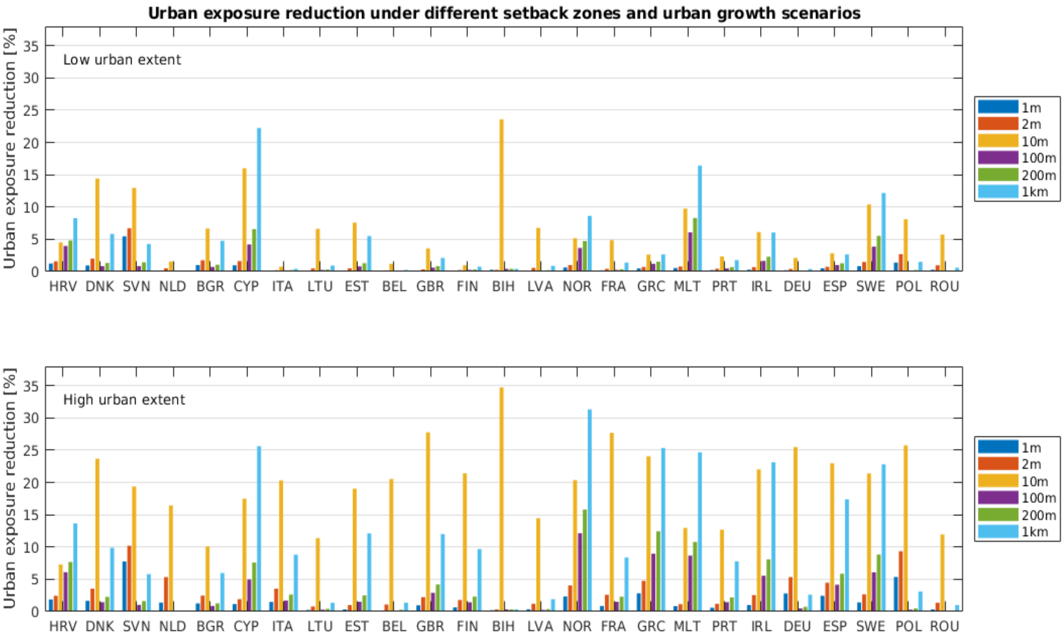

Supplementary Table 1: Retreat potential: The urban areas found in the various setback zones in 2012 [according to CORINE]. The totals are given in km². Additionally, we calculated the proportion of urban areas in different setback zones to each nation's overall urban area.

Country [ISO]		100m		200m		300m		1km		1m		2m		10m		Total
		Urban area in 100m distance to the coast [km²]	Percentage of the total urban area [%]	Urban area in 200m distance to the coast [km²]	Percentage of the total urban area [%]	Urban area in 300m distance to the coast [km²]	Percentage of the total urban area [%]	Urban area in 1km distance to the coast [km²]	Percentage of the total urban area [%]	Urban area below 1m elevation [km²]	Percentage of the total urban area [%]	Urban area below 2m elevation [km²]	Percentage of the total urban area [%]	Urban area below 10m elevation [km²]	Percentage of the total urban area [%]	Total urban area per country [km²]
1	NOR	362	13	521	18	666	24	1174	41	40	1	69	2	328	12	2830
2	MLT	10	10	14	15	18	19	37	39	0	0	0	0	5	6	94
3	DNK	172	5	270	8	374	11	901	25	68	2	167	5	997	28	3555
4	GRC	258	6	382	9	496	12	956	23	30	1	73	2	502	12	4242
5	IRL	69	4	109	7	150	9	345	21	6	0	16	1	213	13	1677
6	HRV	137	6	192	9	240	11	391	19	14	1	28	1	131	6	2112
7	SWE	318	5	467	7	610	9	1240	18	46	1	97	1	827	12	6762
8	CYP	15	3	24	5	33	6	80	15	2	0	5	1	39	8	524
9	EST	20	2	31	3	42	4	114	12	1	0	3	0	100	10	982
10	ESP	304	2	476	4	648	5	1471	11	45	0	101	1	818	6	12927
11	PRT	56	2	91	3	132	4	385	11	7	0	14	0	169	5	3509
12	ITA	397	2	601	4	809	5	1708	10	104	1	281	2	1790	11	16489
13	GBR	364	2	583	3	820	4	2171	10	36	0	97	0	2093	10	21067
14	FIN	87	2	135	3	184	4	446	10	25	1	56	1	564	12	4563
15	LVA	10	1	18	1	27	2	78	6	4	0	16	1	321	24	1318
16	FRA	301	1	472	1	652	2	1592	5	35	0	114	0	1658	5	32454
17	NLD	34	1	51	1	68	1	164	3	355	7	1321	25	3417	64	5367
18	BEL	27	0	42	1	59	1	165	3	1	0	21	0	956	15	6386
19	SVN	3	0	5	1	6	1	13	2	2	0	3	0	10	1	691
20	BGR	21	0	34	1	46	1	91	2	4	0	9	0	59	1	5300
21	DEU	42	0	73	0	106	0	291	1	95	0	223	1	1578	5	33368
22	POL	17	0	27	0	38	0	104	1	33	0	78	0	335	2	18913
23	ROU	16	0	23	0	30	0	70	1	6	0	20	0	145	1	13084
24	LTU	1	0	2	0	3	0	11	1	2	0	5	0	59	3	2186
25	BIH	0	0	0	0	1	0	1	0	0	0	0	0	3	0	850

Supplementary Figure 2: Increase in urban fabric in the E-LECZ in 2100. Comparison of a No Setback zone and 100m setback zone scenario under a high urban growth scenario.

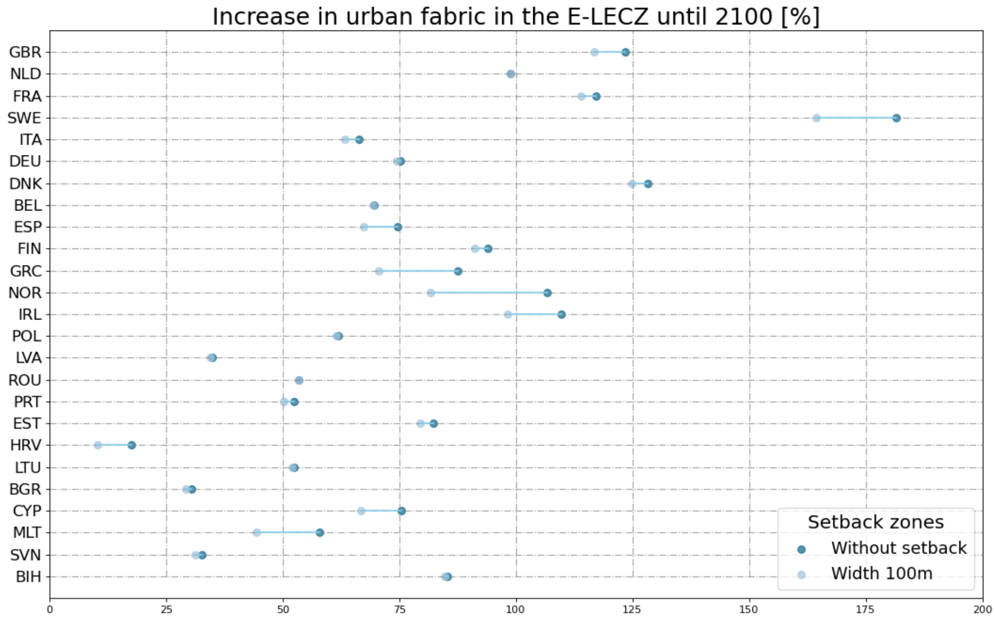

Supplementary Figure 3: Increase in urban fabric in the E-LECZ in 2100. Comparison of a no Setback zone and 10m setback zone scenario under a high urban growth scenario.

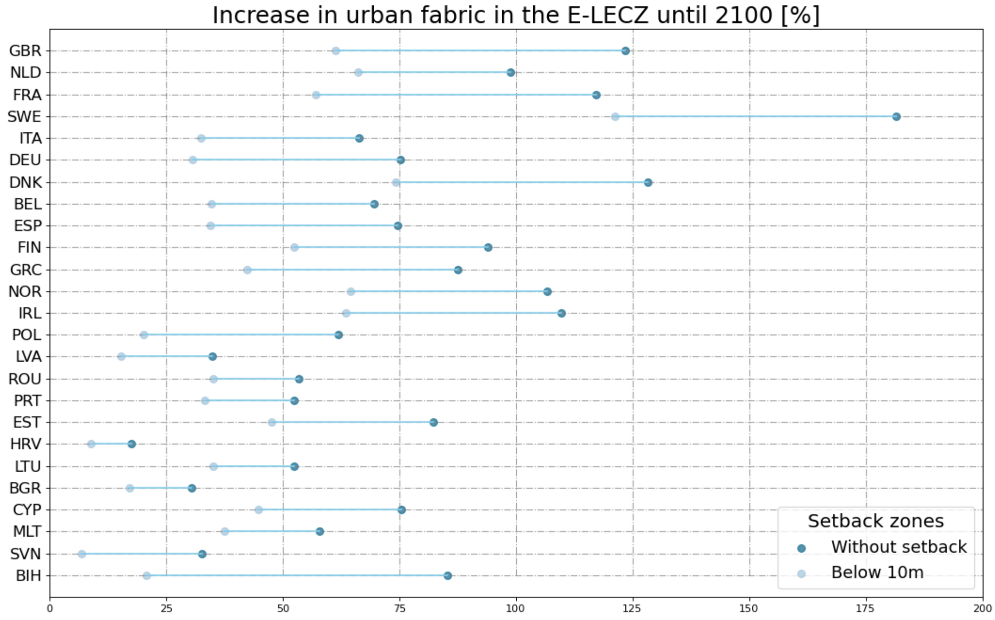

Supplementary Figure 4: Increase in urban fabric in the E-LECZ in 2100. Comparison of a no Setback zone and 1km setback zone scenario under a high urban growth scenario.

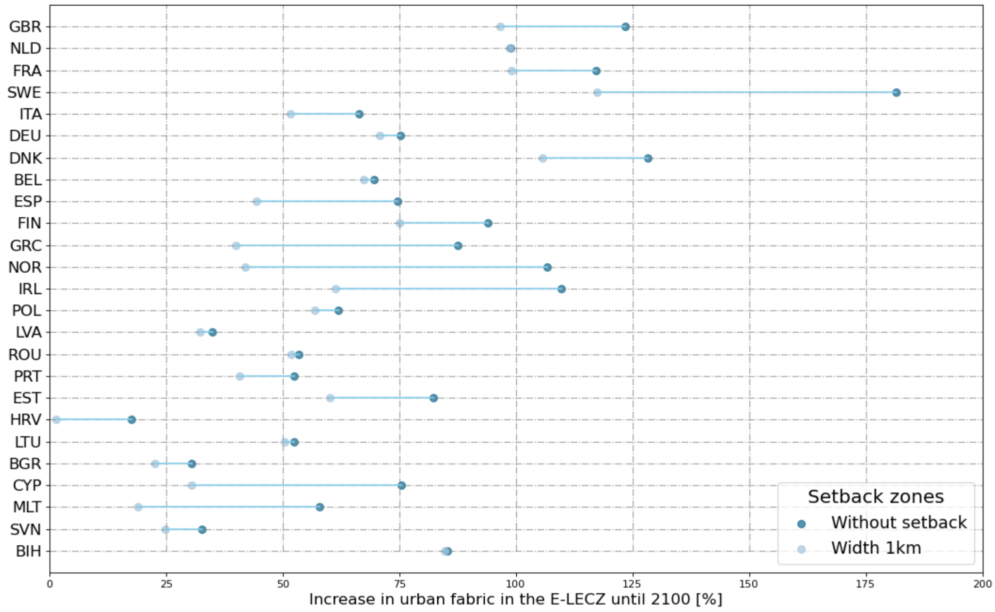

Supplementary Figure 5: Increase in urban fabric in the E-LECZ in 2100. Comparison of a no Setback zone and 10m setback zone scenario under a low urban growth scenario.

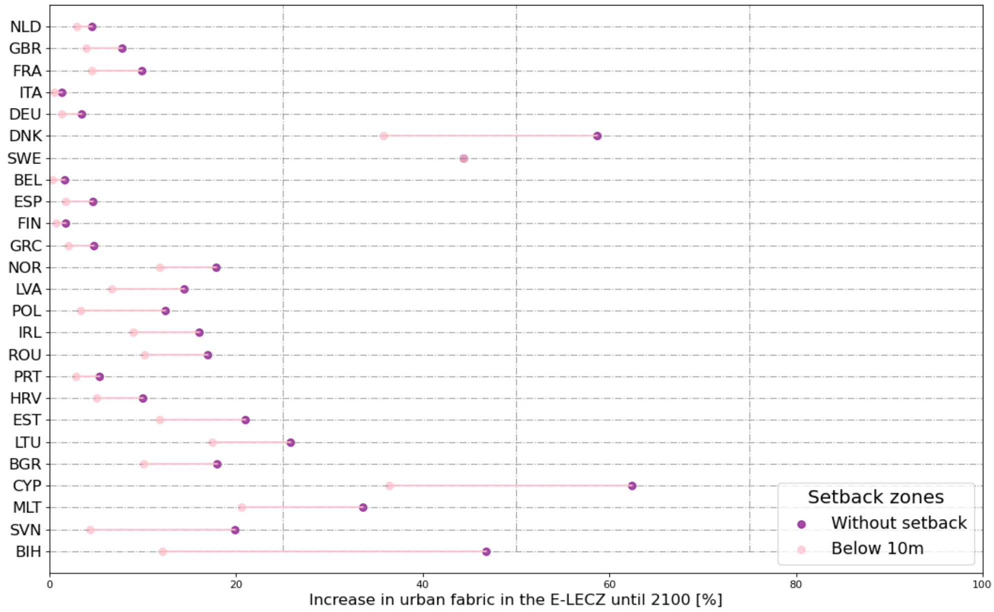

Supplement: Supplementary file 1 — Supplementary Information. [file 41598_2023_32059_MOESM1_ESM.pdf]
